# Sex differences in the relationship between obesity and hypertension in Japan: a large population-based cross-sectional study

**DOI:** 10.1038/s41440-025-02187-y

**Published:** 2025-03-19

**Authors:** Tomomi Nagahata, Nagako Okuda, Kozo Tanno, Yuki Yonekura, Aya Higashiyama, Hirokazu Taniguchi, Koki Kosami, Akira Okayama

**Affiliations:** 1 Division of Applied Life Sciences, Graduate School of Life and Environmental Sciences, Kyoto Prefectural University, 1-5 Hangi-cho, Shimogamo, Sakyo-ku, Kyoto, 606-8522 Japan; 2 Department of Hygiene and Preventive Medicine, Iwate Medical University, 1-1-1 Idaidori, Yahaba-cho, Shiwa-gun, Iwate, 028-3694 Japan; 3 Department of Nursing Informatics, Graduate School of Nursing Science, St. Luke’s International University, 10-1 Akashi-cho, Chuo-ku, Tokyo, 104-0044 Japan; 4 Department of Hygiene, Wakayama Medical University, 811-1 Kimiidera, Wakayama-shi, Wakayama, 641-8509 Japan; 5 Division of Public Health, Center for Community Medicine, Jichi Medical University, 3311-1 Yakushiji, Shimotsuke-shi, Tochigi, 329-0498 Japan; 6 Research Institute of Strategy for Prevention, 10-14 Tomizawa-cho, Nihonbashi, Chuo-ku, Tokyo, 103-0006 Japan

**Keywords:** Obesity, Hypertension, Japanese, Sex, Epidemiology

## Abstract

We examined the effect of sex on the relationship between obesity and hypertension among Japanese people, who generally have a lower prevalence of obesity than Westerners. We analyzed the results of specific health checkups for Japanese aged 40–74 years (688,306 men and 891,191 women) obtained in 2011. The participants were divided into four age groups (40–49, 50–59, 60–69, and 70–74 years) and five body mass index (BMI) categories (≤ 24.9 [non-overweight/obesity], 25.0–26.9 and 27.0–29.9 [overweight], 30.0–34.9 and ≥ 35.0 kg/m^2^ [obesity]). The odds ratio for hypertension in each BMI category was calculated using normal weight as the reference. The prevalence of hypertension was 26.8%–65.5% for men and 17.6%–53.6% for women in the overweight or obesity categories in the 40–49 age group, and 72.0%–88.7% for men and 70.1%–90.6% for women in the 70–74 age group. In women aged 40–49, the prevalence of hypertension in each BMI category was approximately 10% lower than that in men, but there was almost no difference between men and women in the 70–74 age group. On the other hand, the odds ratio for hypertension was higher in women than in men across all BMI categories and age groups. A stronger relationship between obesity and hypertension was observed in women than in men in all age groups. Japanese women have not been the target of studies for obesity, but more attention should be paid to Japanese women with obesity.

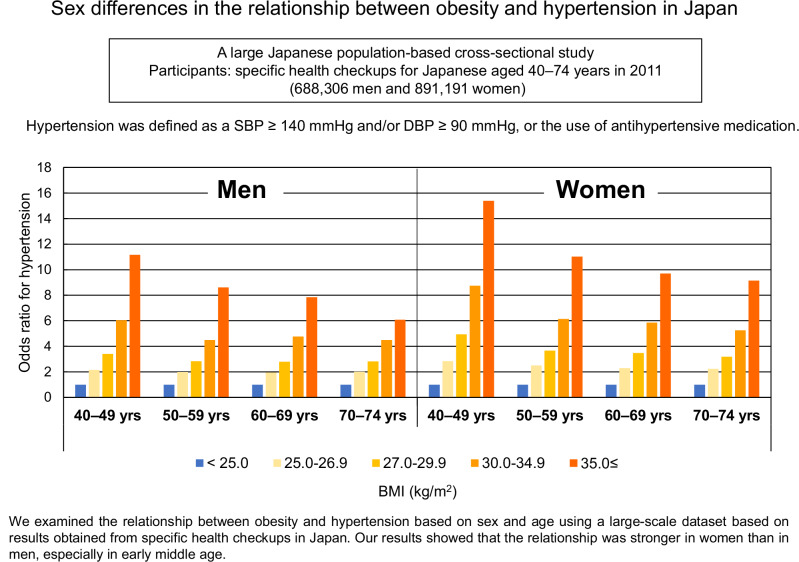

## Introduction

Cardiovascular diseases (CVDs) are the leading cause of death in Japan [1], and hypertension is one of the important risk factors for CVDs in Japan and many other countries [2–4]. The prevalence of hypertension among adults is approximately 40%–60% in Japan, as well as in the US and European countries [5–7]. It is particularly high among the elderly, above 50% in Japanese men and women aged 60 years and over [8].

Obesity is a major lifestyle-related factor for hypertension [9–12], but obesity has not been examined in detail; it was only used as an adjustment factor in many epidemiological studies in Japan [4, 13], mainly because of the low prevalence of obesity compared with that in Western countries. The proportions of overweight (OW, 25.0 kg/m^2^ ≤ body mass index (BMI) < 30.0 kg/m^2^) or obesity (OB, BMI ≥ 30 kg/m^2^) (OW/OB) in Japanese men and women were 33.0% and 22.3%, respectively, in the National Health and Nutrition Survey Japan (NHNSJ) 2019 [6], while the World Health Organization reported that 59% of adults worldwide were OW/OB in 2022 [14].

However, the percentage of OW/OB Japanese adults has been increasing in recent years, especially among men [6, 15], and this health issue has become increasingly apparent. Following the trend, specific health checkups (SHC) and specific health guidance (SHG) have been provided since 2008 with a legal basis, to prevent metabolic syndrome (Mets) associated with obesity [15]. Under this system, health insurers are obligated to provide SHCs for insured persons aged 40–74 years and SHGs for those classified as OW/OB with a risk of developing CVDs according to the SHC results. Reflecting the percentage of OW/OB, many more men than women are identified as eligible for SHG; the number of SHG persons is approximately 4 times more for men than for women in data registered with the Ministry of Health, Labor and Welfare in 2022 [16]. Thus, more attention has been paid to men and less to women in the field of health education on obesity in Japan.

Although obesity is a critical risk factor for hypertension, no studies have comprehensively examined whether sex influences the relationship between obesity and hypertension. Considering that the age groups with higher proportions of obesity and hypertension differ between men and women [17, 18], the strength of the relationship between obesity and hypertension may also differ by age group. In this study, we used a large-scale dataset of body mass and blood pressure obtained from SHCs in Japan and examined the relationship between obesity and hypertension based on sex and age.

Point of view

- **Clinical relevance** Explanations should be given to middle-aged women with normal blood pressure but an increasing weight that they are at higher risk of developing hypertension, and they should monitor their weight and BP.
- **Future direction** It is important to provide specific information to women around menopause, warning that they should be careful about weight gain and elevated blood pressure.
- **Consideration for the Asia population** The results of this study, which showed a stronger relationship between obesity and hypertension in women than in men, may applies to other Asian countries with less obesity people and comparable prevalence of hypertension compared to Western countries, so further research is needed.

## Methods

### Dataset

With the collaboration of 152 health insurers (143 national health insurers and 9 health insurance associations) and 3 medical institutions performing SHG, we obtained an anonymized SHC dataset comprising results collected in 2011 for 2,490,660 individuals (1,101,083 men and 1,389,577 women). The details of the data collection have been previously described [19, 20].

For the current analysis, 911,163 participants were excluded because of missing data on age, BMI, systolic blood pressure (SBP), diastolic blood pressure (DBP), and information on the use of antihypertensive medication. Those whose ages were < 40 or ≥ 75 were also excluded. The remaining 1,579,497 (688,306 men and 891,191 women) participants were included in the analysis.

This study was performed with approval from the ethics committee at the Research Institute of Strategy for Prevention (No. 4-2) and from Kyoto Prefectural University (No. 257).

### Statistical analysis

Hypertension was defined as a SBP ≥ 140 mmHg and/or DBP ≥ 90 mmHg, or the use of antihypertensive medication. The participants were divided into four age groups (40–49, 50–59, 60–69, and 70–74 years) and five BMI categories (≤ 24.9, 25.0–26.9, 27.0–29.9, 30.0–34.9, and ≥ 35.0 kg/ m^2^). Participants with BMI ≤ 24.9 kg/m^2^ were labeled non-OW/OB because the Japan Society for the Study of Obesity defines BMI ≥ 25.0 kg/m^2^ as obesity. The BMI ranges of 25.0–26.9 and 27.0–29.9 kg/m^2^ were labeled OW, and the ranges of 30.0–34.9 and ≥ 35.0 kg/m^2^, which correspond to WHO class 1 obesity and class 2 obesity, respectively, were labeled OB. All analyses were performed separately for men and women.

First, the proportion of participants in each BMI category was calculated, and then the prevalence of hypertension was calculated for each age group, BMI category, and sex. Cochran’s Armitage test was used to examine trends across the age groups. The odds ratio (OR) and relative prevalence (RP) for hypertension in each BMI category were calculated using the non-OW/OB group as a reference. The sex- and age-specific population attributable fractions (PAF) for hypertension in each BMI category were calculated using the RP of hypertension. The following formula was used to calculate the PAF [21], where *PF* is the population fraction (%) for each BMI category.

$${{{\rm{PAF}}}}( \% )=[({{{\rm{RP}}}}-1)/{{{{\rm{RP}}}}}]\times {PF}$$

All analyses were performed using SPSS ver. 29.0 (IBM Corporation, Tokyo, Japan). *P* values of less than 0.05 (two-sided) were considered to indicate statistical significance.

## Results

Distributions of the participants across the five BMI categories in each age group are shown in Table 1. The proportions of OW/OB were more than twice as high for men than for women in the 40–49 age group, but this difference was smaller or partially reversed in older groups. The proportions of OW/OB in the 40–49 and 70–74 age groups were 33.7% and 26.0% for men and 15.7% and 21.6% for women, respectively. In the 70–74 age group, the proportion of OB was 1.7% for men and 2.8% for women. For men, the proportion of OW was lower in the older age group than in the younger group (trend-*P* < 0.001); however, for women, was higher in the older age group (trend-*P* < 0.001).

**Table 1:** Prevalence of overweight or obesity ranges, and hypertension, among Japanese men and women aged 40–74 years (688,306 men and 891,191 women, specific health checkup data in 2011)

	Total		Non-OW/OB		OW		OB		OW				OB			
BMI (kg/m^2^)			< 25.0		25.0–29.9		30.0 ≤		25.0–26.9		27.0–29.9		30.0–34.9		35.0 ≤	
	n	(%)	n	(%)	n	(%)	n	(%)	n	(%)	n	(%)	n	(%)	n	(%)
Number and proportion of participants (%)																
Men																
40–49 yrs	118,848	(100)	78,820	(66.3)*	32,675	(27.5)*	7353	(6.2)*	19,008	(16.0)	13,667	(11.5)*	6150	(5.2)*	1203	(1.0)*
50–59	108,761	(100)	73,050	(67.2)	31,124	(28.6)	4587	(4.2)	19,034	(17.5)	12,090	(11.1)	4020	(3.7)	567	(0.5)
60–69	280,967	(100)	204,787	(72.9)	70,360	(25.0)	5820	(2.1)	46,710	(16.6)	23,650	(8.4)	5402	(1.9)	418	(0.1)
70–74	179,730	(100)	133,008	(74.0)	43,589	(24.3)	3133	(1.7)	29,432	(16.4)	14,157	(7.9)	2939	(1.6)	194	(0.1)
Women																
40–49 yrs	104,854	(100)	88,384	(84.3)*	12,590	(12.0)*	3880	(3.7)*	7171	(6.8)*	5419	(5.2)*	3040	(2.9)*	840	(0.8)*
50–59	131,893	(100)	108,336	(82.1)	19,365	(14.7)	4192	(3.2)	11,495	(8.7)	7870	(6.0)	3503	(2.7)	689	(0.5)
60–69	424,679	(100)	343,295	(80.8)	70,382	(16.6)	11,002	(2.6)	43,677	(10.3)	26,705	(6.3)	9652	(2.3)	1350	(0.3)
70–74	229,765	(100)	180,103	(78.4)	43,270	(18.8)	6392	(2.8)	26,828	(11.7)	16,442	(7.2)	5704	(2.5)	688	(0.3)
Number and prevalence of hypertensives (%)																
Men																
40–49 yrs	25,486	(21.4)	11,460	(14.5)*	10,120	(31.0)*	3906	(53.1)*	5098	(26.8)*	5022	(36.7)*	3118	(50.7)*	788	(65.5)*
50–59	44,118	(40.6)	24,324	(33.3)	16,555	(53.2)	3239	(70.6)	9481	(49.8)	7074	(58.5)	2779	(69.1)	460	(81.1)
60–69	154,453	(55.0)	101,528	(49.6)	48,104	(68.4)	4821	(82.8)	30,759	(65.9)	17,345	(73.3)	4451	(82.4)	370	(88.5)
70–74	109,773	(61.1)	74,794	(56.2)	32,301	(74.1)	2678	(85.5)	21,202	(72.0)	11,099	(78.4)	2506	(85.3)	172	(88.7)
Women																
40–49 yrs	10,548	(10.1)	6166	(7.0)*	2728	(21.7)*	1654	(42.6)*	1264	(17.6)*	1464	(27.0)*	1204	(39.6)*	450	(53.6)*
50–59	35,641	(27.0)	24,075	(22.2)	8811	(45.5)	2755	(65.7)	4786	(41.6)	4025	(51.1)	2232	(63.7)	523	(75.9)
60–69	187,328	(44.1)	134,127	(39.1)	44,417	(63.1)	8784	(79.8)	25,970	(59.5)	18,447	(69.1)	7621	(79.0)	1163	(86.1)
70–74	129,118	(56.2)	92,203	(51.2)	31,464	(72.7)	5451	(85.3)	18,817	(70.1)	12,647	(76.9)	4828	(84.6)	623	(90.6)

Hypertension was defined as follows: systolic blood pressure ≥ 140, or diastolic blood pressure ≥ 90, or using antihypertensives

*BMI* body mass index, *OW* overweight, *OB* obesity

*p < 0.05 obtained from trend analysis across age categories

The prevalences of hypertension are also shown in Table 1. In the 40–49 age group, the prevalences were approximately 10% higher for men than for women in the same BMI categories. The prevalences of hypertension in OW and OB were 31.0% and 53.1% for men but 21.7% and 42.6% for women, respectively. In each BMI category, the prevalence of hypertension was higher in the older age group than in the younger age group (trend-*P* < 0.001) for both sexes; however, the difference between men and women was smaller in the older age group. In the 70–74 age group, the prevalences of hypertension in OW and OB were 74.1% and 85.5% for men and 72.7% and 85.3% for women, respectively.

In each age group, ORs for hypertension were higher in higher BMI categories for both sexes (Table 2), and they were higher in the younger age group than in the older age group in each BMI category. In most of the age groups and BMI categories, the ORs for hypertension were significantly higher for women than for men because of non-overlapping 95% confidence intervals. All ORs in OW categories were significantly higher for women than for men in all age groups. In the OB categories, all ORs were significantly higher for women than for men in the 40–49 and 50–59 age groups. In the older group, only the OR in the 30.0 ≤ BMI ≤ 34.9 kg/m^2^ category aged 60–69 was significantly higher for women than for men.

**Table 2:** Odds ratios of hypertension for overweight or obesity participants with respect to non–overweight/obesity participants among Japanese men and women in different age groups (688,306 men and 891,191 women, specific health checkup data in 2011)

	non–OW/OB	OW				OB			
BMI (kg/m^2^)	< 25.0	25.0–26.9		27.0–29.9		30.0–34.9		35.0 ≤	
	OR	OR	(95% CI)	OR	(95% CI)	OR	(95% CI)	OR	(95% CI)
Men									
40–49 yrs	1	2.15	(2.07–2.24)	3.41	(3.28–3.55)	6.04	(5.73–6.38)	11.16	(9.89–12.59)
50–59	1	1.99	(1.92–2.05)	2.83	(2.72–2.94)	4.49	(4.19–4.80)	8.61	(6.97–10.63)
60–69	1	1.96	(1.92–2.00)	2.80	(2.71–2.88)	4.76	(4.44–5.11)	7.84	(5.80–10.59)
70–74	1	2.01	(1.95–2.06)	2.82	(2.71–2.94)	4.50	(4.07–4.99)	6.09	(3.90–9.49)
Women									
40–49 yrs	1	2.85	(2.67–3.05)	4.94	(4.62–5.27)	8.74	(8.09–9.45)	15.39	(13.40–17.66)
50–59	1	2.50	(2.40–2.60)	3.66	(3.50–3.84)	6.15	(5.73–6.59)	11.03	(9.25–13.14)
60–69	1	2.29	(2.24–2.33)	3.48	(3.39–3.58)	5.85	(5.57–6.15)	9.70	(8.31–11.32)
70–74	1	2.24	(2.18–2.30)	3.18	(3.06–3.30)	5.25	(4.89–5.65)	9.14	(7.08–11.80)

Hypertension was defined as follows: systolic blood pressure ≥ 140, or diastolic blood pressure ≥ 90, or using antihypertensives

*BMI* body mass index, *OW* overweight, *OB* obesity, *OR* odds ratio, *CI* confidence interval

The RP exhibited a similar pattern to the ORs (Table 3). Reflecting the higher proportion of OW than OB, PAFs were higher in the OW categories than in the OB categories for both men and women (Table 3). Furthermore, the PAFs were higher in the younger age group than in the older age group; PAFs of OW/OB in the 40–49 and 70–74 age groups were 18.7% and 6.4% for men and 11.0% and 6.7% for women, respectively.

**Table 3:** Relative prevalence (RP) and population attributable fraction (PAF) of hypertension for overweight or obesity participants among Japanese men and women in different age groups (688,306 men and 891,191 women, specific health checkup data in 2011)

		non-OW/OB	OW		OB	
	BMI (kg/m^2^)	< 25.0	25.0–26.9	27.0–29.9	30.0–34.9	35.0 ≤
Men						
40–49 yrs	RP	1	1.84	2.53	3.49	4.51
	PAF (%)	-	7.3	6.9	3.7	0.8
50–59	RP	1	1.5	1.76	2.08	2.44
	PAF (%)	-	5.8	4.8	1.9	0.3
60–69	RP	1	1.33	1.48	1.66	1.79
	PAF (%)	-	4.1	2.7	0.8	0.1
70–74	RP	1	1.28	1.39	1.52	1.58
	PAF (%)	-	3.6	2.2	0.6	0
Women						
40–49 yrs	RP	1	2.53	3.87	5.68	7.68
	PAF (%)	-	4.1	3.8	2.4	0.7
50–59	RP	1	1.87	2.3	2.87	3.42
	PAF (%)	-	4.1	3.4	1.7	0.4
60–69	RP	1	1.52	1.77	2.02	2.2
	PAF (%)	-	3.5	2.7	1.1	0.2
70–74	RP	1	1.37	1.5	1.65	1.77
	PAF (%)	-	3.2	2.4	1	0.1

*BMI* body mass index, *OW* overweight, *OB* obesity

## Discussion

**Fig. 1:**
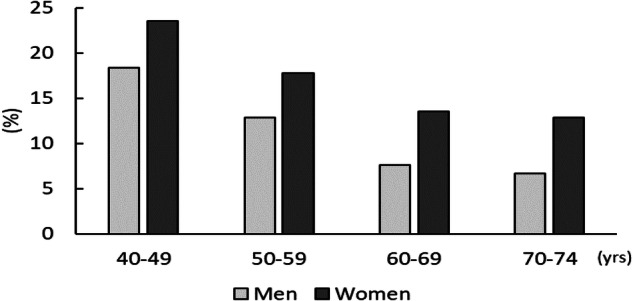
Proportion of obesity among overweight/obesity participants for each sex and age group

In this study, the ORs for hypertension were higher in women than in men in each BMI category, indicating a stronger relationship between obesity and hypertension in women than in men. Although the PAFs were higher in men based on the higher proportion of OW/OB, showing the importance of taking measures against obesity in men, the stronger relationship in women indicated by the higher ORs revealed a similar need for hypertension and obesity prevention in women. In addition, the proportion of OB among OW/OB was higher for women than for men in all age groups (Fig. 1), despite the lower percentage of OW/OB in women than in men. Our findings have shown that a significant number of women may require obesity prevention for hypertension and its complications, suggesting that more research focused on effective obesity prevention measures among Japanese women is necessary.

A previous study in the US reported that the effect of BMI on CVD risk had been stronger in women than in men [22]. Although the proportion of obesity is quite low in Japanese women, we observed a stronger relationship between obesity and hypertension in women than in men, especially in early middle age. In the 40–49 age group, the ORs for hypertension of the OB category were 6–11 in men and 8–15 in women, and they were 4–6 in men and 5–9 in women in the 70–74 age group. A previous study on secular trends in the relationship between obesity and hypertension using the results of the NHNSJ from 1980 to 2010 showed that the age-standardized ORs for hypertension in OW/OB adjusted with lifestyle factors increased during this period in both men and women (from 1.94 to 2.82 in men and from 2.37 to 3.48 in women) [23], suggesting the growing importance of obesity in the development of hypertension. However, age-specific ORs were not calculated because of the small sample sizes. Age-specific analysis may provide useful information for public health policy, especially in planning individual health guidance programs.

In the present analysis, the age-specific prevalence of hypertension in the OB category ranged from 53% to 85% for men and from 42% to 85% for women. In a study using results from the US National Health and Nutrition Examination Survey in 1999–2010, the age-standardized prevalence of hypertension in US adults with OB was around 35% [24]. Although it is difficult to compare the prevalence between the two populations with different age structures, the prevalence in the OB category may be higher in Japan than in the US. A previous study considering participants of various ethnicities in Canada reported that East Asians were at higher risk of developing hypertension associated with increased BMI than Caucasians, particularly among women in the OW range [25]. Obesity prevention may be more effective as a measure to prevent hypertension in Japan than in Western countries, and in women than in men.

The prevalence of hypertension in the non-OW/OB range in each age group was higher for men (14%–56%) than for women (7%–51%) in this study, and the ORs for hypertension in the OW/OB range were higher for women than for men. In addition to obesity, several other lifestyle factors cause hypertension, namely high salt intake, excessive alcohol consumption, insufficient intake of vegetables and fruit, and low physical activity levels [26]. According to NHNSJ 2019, the average salt intake of adults was shown to be 10.8 g/day for men and 9.1 g/day for women, and 14.7% of men and 9.8% of women had drinking habits affecting lifestyle-related diseases [6].

In this analysis, the proportion of OW/OB was lower in older men than in younger men and higher in older women than in younger women. It should be noted that the percentage trend of obesity by age group in men and women in this study includes the cohort effects. According to the NHNSJ, the average BMI by age group has recently been increasing in men and decreasing in women [6, 17, 27]. Although class 2 obesity is rare in Japanese people [28], we could show the prevalence using the current dataset, which was sufficiently large. Given the increasing trend of the average BMI in men, it may be important to monitor the trend of class 2 obesity when considering future prevention strategies.

Those who have undergone SHCs are stratified as having Mets or at risk of developing Mets according to the SHC results. The proportion of participants who were OW/OB and hypertensive (i.e., those who were likely to be eligible for SHG), was higher for men than for women, especially in the early middle-aged participants in the present analysis, similar to the registered data [16]. The difference between men and women was smaller in their 50 s and above. Because more men have participated in SHGs than women, most studies examining the effects of SHGs have been conducted with male participants [29, 30], and few studies with women. Development of obesity prevention programs for early middle-aged women should be promoted and the effectiveness of such programs should be tested. After 50 years of age, specifically after menopause, many women experience body weight increase and blood pressure elevation. Under the SHC system, those who are normal weight are only given a pamphlet about lifestyle-related disease, along with each SHC result. The same pamphlets are given to men and women regardless of their age, but it may be effective to provide specific contents to peri-menopausal women (aged 40–50), clarifying that they should be careful about weight gain and elevated blood pressure, even if they currently have a normal weight and normal blood pressure. Such education programs may be effective in the prevention of hypertension, with potentially greater public health benefits in Japan.

This study has several limitations. First, this dataset was obtained from the results of SHCs performed by a limited number of health insurance associations, and the age structure of the sample population differed from that of Japan overall. Therefore, we considered the body mass distribution and the prevalence of hypertension in each age group and did not show those values for all participants of all age groups. Second, the data were obtained from those who had undergone SHCs, and thus the participants may have been relatively healthier than the general population. The participants of this study received health checkups given by insurers, and many of them regularly received checkups; they received the results and had chances to improve their lifestyles or seek medical treatment if necessary. Therefore, the proportion of OW/OB, as well as the prevalence of hypertension, may be lower in this population than in the general population, which includes those who had not received the checkups. Third, we presented the crude relationships between the BMI category and hypertension and did not adjust the other lifestyle factors because the SHC data did not include sufficient information for their adjustment. In a cross-sectional study in Japan, the OR for hypertension associated with obesity adjusted for drinking habits and other lifestyle factors were calculated, and the adjusted OR was lower than the non-adjusted OR while maintaining a significant difference [31]. Another cross-sectional study in Japan also reported a higher contribution of obesity to hypertension in women than men after adjustment for lifestyle factors [32]. The ORs for hypertension from obesity are likely to remain higher in women than in men in the present study, even after adjustment.

### Perspective of Asia

Obesity is the major lifestyle-related factor for hypertension [9–12]. In Asian countries other than Japan, the percentage of obesity people is also apparently lower than in the US and European countries [33], but the prevalence of hypertension is at a comparable level [5]. Blood pressure elevation is strongly associated with age, and weight increases often occur prior to the development of hypertension. By examining the relationship between physique and blood pressure by gender and age group in other Asian countries, it may be possible to propose effective measures for preventing hypertension there.

## Conclusion

This study examined the relationship between obesity and hypertension based on sex and age group using large-scale SHC data from a Japanese population. In conclusion, the results showed that the relationship was stronger in women than in men, despite the small number of OW/OB, especially in early middle age, suggesting that obesity control as hypertension prevention measures may be more effective in early middle-aged Japanese women. Overall, few studies have focused on obesity in women, and further studies are necessary to accumulate evidence regarding the association between obesity and metabolic factors in women.
